# Critical Care in the Emergency Department: An assessment of the length of stay and invasive procedures performed on critically ill ED patients

**DOI:** 10.1186/1757-7241-17-47

**Published:** 2009-09-24

**Authors:** Robert S Green, Janet K MacIntyre

**Affiliations:** 1 Department of Emergency Medicine, Dalhousie University, Halifax, Nova Scotia, Canada; 2 Department of Medicine, Division of Critical Care Medicine, Dalhousie University, Halifax, Nova Scotia, Canada

## Abstract

**Introduction:**

Critically ill patients commonly present to the ED and require aggressive resuscitation. Patient transfer to an ICU environment in an expedient manner is considered optimal care. However, this patient population may remain in the ED for prolonged periods of time. The goal of this study is to describe the ED length of stay, and the invasive procedures performed in critically ill ED patients.

**Methods:**

This is a retrospective medical record review of all patients who presented to the study center over a 1 year period. Patient demographic data, in addition to the times of ED presentation and ICU admission were recorded. Invasive procedures performed in the pre-hospital, ED and the initial 24 hours of ICU care were also recorded.

**Results:**

Overall, 178 patients' required direct admission to an ICU from the ED, with a mortality rate of 21.9%. The median LOS in the ED for critically ill patients requiring ICU admission was 4.9 h (mean 6.5 h, range 1.4-28.2 h). Seventy percent of patients (125,178, 70.2%) required endotracheal intubation with the majority (118/125, 94.4%) being performed in the ED (80/125, 64.0%) or the prehospital setting (38/125, 30.4%). Central venous access was obtained in 56/178 patients (31.5%), with 17.9% (10/56) completed in the ED. Similarly, arterial catheters were inserted in 99/178 patients (55.6%) with 14.1% (14/99) inserted in the ED.

**Conclusion:**

Critically ill patients are managed in the emergency department for a significant length of time. Although the majority of airway intervention occurs in the prehospital setting and ED, relatively few patients undergo invasive procedures while in the emergency department.

## Background

Critically ill patients are common in emergency medicine and require early and aggressive care to optimize outcomes. [1-5] Emergency medicine (EM) physicians are challenged to provide expert care to severely ill patients while balancing the needs of other patients within the emergency department (ED). [2,3,6] Unfortunately, increasing numbers of critically ill patients are presenting to the ED and are managed for prolonged periods of time despite requiring admission to an intensive care unit (ICU). [1,3,7-11]

Data on the management of critically ill patients in the ED is incomplete. The primary objective of this study is to determine the length of stay of critically ill patients receiving care in a tertiary care adult emergency department. The secondary objective is to describe the invasive procedures performed in the ED phase of care.

## Methods

This study was a retrospective chart review that included all patients presenting to the Queen Elizabeth II Health Sciences Center in Halifax, Nova Scotia, Canada and admitted directly to the one of two mixed medical/surgical/neurosurgical intensive care units from the ED over a one year period (January 1, 2002 through December 31, 2002). The Queen Elizabeth II Health Sciences Center ED is an adult (age ≥17 years) tertiary care ED with approximately 70,000 patient visits per year. Inclusion criteria was any patient who was assessed and managed by the ED physician and was subsequently admitted to one of two Intensive Care Units. Exclusion criteria included patients under 17 years, patients transferred to the ED from another hospital, patients managed by the multi-disciplinary trauma team (and therefore may not have been managed by an ED physician), or patients requiring surgical intervention prior to ICU admission.

Patients were identified by manual review of both ED and ICU admission records. A standardized electronic data abstraction form was developed by the investigators. Approximately 10% of data abstraction was reviewed by both investigators to ensure data reliability. Any discrepancy in data was resolved by consensus. All available data in the medical record was recorded into the database. Missing data that was unavailable in the medical record were also noted and data analysis was based on available data. Procedures not recorded in the medical record were recorded as not being preformed.

Data was collected for 3 phases of medical care: the prehospital phase, ED phase and the initial 24 hours after ICU admission and included patient demographics, ED diagnosis, Canadian Triage Acuity score (CTAS), critical care procedures performed, and the ED and hospital LOS. CTAS is a triage tool developed in conjunction with the Canadian Association of Emergency Medicine to enable ED patient care prioritization, and ranges from CTAS 1 (critically ill) to CTAS 5 (non-emergent). [12] The emergency department length of stay (LOS) was defined as the time from ED triage to transfer to ICU, and hospital LOS was defined as the time from hospital admission to patient discharge. The critical care procedures recorded were endotracheal intubation (ETI), central venous catheter (CVC) and arterial cannulation (AC), and chest tubes insertion.

The data was analyzed using descriptive statistics. Mean and median values and frequencies were calculated. The study was approved by the Research Ethics Board of the Queen Elizabeth II Health Sciences Center, Halifax, Nova Scotia, Canada.

## Results

During the study period, 68,765 patients presented to the ED and 178 patients met inclusion criteria (ICU admission rate 0.26%). The median age of the study population was 55 years and 59.6% were male (Table 1). The in-hospital mortality rate of the study population was 21.9% (39/178). Patients who survived (139/178) were discharged home (111/178, 62.3%) or to long term care or other facilities (26/178, 14.6%).

**Table 1:** Patient Demographics

General	a) Age	Mean 57.9 years	Median 55 years	Range 16-89 years	
					
	b) Sex	Male 106 (59.6%)	Female 72 (40.4%)		
					
CTAS@	CTAS Score Recorded in chart: 70/178 (39.3%)	CTAS 1 11/70 (15.7%)	CTAS 2 39/70 (55.7%)	CTAS 3 20/70 (28.6%)	CTAS 4 or 5 0/70
					
	Mortality per CTAS	2/11 (18.2%)	8/29 (26.51%)	5/20 (20.0%)	
					
Mortality*	39/178 (21.9%)				
					
LOS	a) ED LOS#	Mean 6.5 h	median 4.9 h	Range 1.4-28.2 h	
					
	b) Hospital LOS$	498.5 h (20.8 days)	216 h (9.0 days)	Range 24-8688 h (1-362 days)	
Discharge Location	Alive: 139/178 (78.1%)	Home 111/178 (62.3%)	Long term care facility% 8/178 (4.5%)	Other% 18/178 (10.1%)	Unknown 2 (1.1%)

@ Canadian Triage Acuity Score

* In-hospital mortality;

# Emergency Department length of stay

$ Hospital length of stay

% Rehabilitation hospital or similar facility

The median LOS in the ED for critically ill patients requiring ICU admission was 4.9 h (mean 6.5 h, range 1.4-28.2 h) and the median hospital LOS was 9 days (mean 20.8 days, range 1-362 days). Seventy patients (70/178, 39.3%) were assigned a CTAS score in the ED, with 11/70 (15.7%) assigned CTAS level 1, 39/70 (55.7%) CTAS level 2 and 20/70 (28.6%) CTAS level 3. The ED diagnosis of critically ill patients varied (Table 2).

**Table 2:** ED diagnosis of critically ill patients

Respiratory System 34/178 (19.1%)	COPD& 9	Asthma 3	Pneumonia 12	Resp Failure NYD 7	Other 3
Unknown_!_ 33/178 (18.5%)					
Central Nervous System 27/178 (15.2%)	CVA 8	Decreased LOC@ 6	ICH# 8	Seizure 4	Other 1
Toxic Ingestion 26/178 (14.6%)					
Trauma 16/178 (9.0%)	Multi-system 11	TBI* 5			
Gastrointestinal System 14/178 (7.9%)	GI Bleed 11	Other 3			
Cardiovascular System 8/178 (4.5%)	Cardiac Arrest 4	ACS$ 1	Pulmonary Edema 2	PE% 1	
Endocrine 7/178 (3.9%)	DKA+ 7				
Genital-urinary System 4/178 (2.4%)	Acute Renal Failure 3	Other 1			
Sepsis-location unknown 3/178 (1.7%)					
Other = 6/178 (3.4%)					

Note: classification is based on primary physiological system affected by patient illness. The majority of patients had multiple physiologic system derangement.

@ Level of consciousness

# Intra-cranial hemorrhage

$ Acute coronary syndrome

% Pulmonary embolus

& Chronic Obstructive Pulmonary Disease

* Traumatic Brain Injury

+ Diabetic ketoacidosis

= Other: epistaxis, chart incomplete (2), suicide attempt, supraglotitis swelling, neck haematoma

! Reason for ICU admission not stated in chart, or multifactorial in nature

The majority of patients received at least one invasive procedure in the ED (Table 3). Of the 178 patients, 125 patients (125,178, 70.2%) required endotracheal intubation during the first 24 hours of their hospital admission. The majority of intubations (118/125, 94.4%) were performed in the ED (80/125, 64.0%) or the prehospital setting (38/125, 30.4%). Central venous access was obtained in 56/178 patients (31.5%). Only 17.9% (10/56) of patients who had a CVC inserted had this procedure performed in the ED. The majority of patients requiring a central venous catheter (30/56, 53.6%) had the CVC inserted within the first 6 hours of admission to the ICU. Similarly, arterial catheters were inserted in 99/178 patients (55.6%) with 14.1% (14/99) inserted in the ED and 71.7% (71/99) inserted in the first 6 hours of ICU admission. Chest tubes insertion was completed in a minority of cases (8/178, 4.5%).

**Table 3:** Invasive procedures completed in patients admitted to an ICU directly from the ED

	**Prehospital** **(n, #)**	**Emergency Department**	ICU <6 h*	**ICU 6-24 h$**
Endotracheal Intubation 125/178 (70.2%)	38/125 (30.4%) Paramedic 38/38	80/125 (64.0%) Staff: 35/80 Resident: 15/80 Paramedic: 4/80 Not recorded: 26/80	4/125 (3.2%) Staff:0/4 Resident: 4/4	3/125 (2.4%) Staff: 0/3 Resident: 3/3
Central venous catheter 56/178 (31.5%)	0	10/56(17.9%) Staff:3/10 Resident: 6/10 Not recorded: 1/10	30/56 (53.6%) Staff: 3/30 Resident:27/30	16/56 (28.6%) Staff: 1/16 Resident: 14/16 Other%:1/16
Arterial Line Catheter 99/178 (55.6%)	0	14/99 (14.1%) Staff: 3/14 Resident: 9/14 Not recorded: 2/14	71/99 (71.7%) Staff:8/71 Resident: 60/71 Other: 3/71	14/99 (14.1%) Staff: 3/14 Resident:8/14 Other:3/14
Chest Tube 8/178 (4.5%)	0	4/8 (50.0%) Staff: 1/4 Resident: 1/4 Not recorded: 2/4	1/8 (12.5%) Staff:0/1 Resident0/1: Other:1/1	3/8 (37.5%) Staff:0/3 Resident3/3:

(n = number of patients with ED diagnosis)

* Procedure completed within 6 hours of ICU admissions

$ Procedure completed >6 hours after ICU admission, but within first 24 hours of ICU admission.

% Other: hospitalist, medical student

## Discussion

We have found that critically ill patients in our study were managed in the ED for 4.9 hours prior to transfer to an ICU. In addition, although the majority of emergent airway management is provided in the ED and pre-hospital setting, other invasive procedures such as central venous catheterization and arterial cannualtion were more commonly preformed after transfer to an ICU setting.

The management of critical illness in the emergency department occurs at a crucial phase in a patient's care, when intervention may significantly improve outcome and survival. [4,5,13] Early and aggressive care for critically ill patients is believed to optimize patient outcomes, as the stabilization of physiological derangements reduces the progression of multi-organ dysfunction. [13-15] However, the ED may not be the optimal location for prolonged or ongoing provision of critical care, as physicians and other health care members have divided priorities in the management of other ED patients. ED physicians and nurses may not possess the skill sets to allow for the provision of optimal care beyond the acute resuscitation. In addition, some ED's may not have the resources available to provide ongoing or prolonged care for critically ill patients, and therefore the rapid transport of patient to an ICU environment is desirable.

The median LOS of patients in our study are similar to previous reports, which range from 4.4-6.2 hours.[1,3,6,7,12] Little data is available for countries other than the USA, and therefore this study highlights a potential global issue. Emergency Department LOS of critically ill patients is likely multifactorial and may include time required for ED diagnosis, resuscitation and necessary investigations. However, other factors such as ED overcrowding, ICU resource availability and local practice patterns may affect ED LOS. Further work focusing on modifiable factors contributing to prolonged ED LOS of critically ill patients would further clarify this issue.

This study has also demonstrated that some invasive procedures are performed frequently in the ED while others are not completed until after admission to the ICU. It is interesting that the majority of airway interventions occurred in the ED prior to ICU admission (94.4%), however relatively few patients underwent invasive procedures such as CVC or AC insertion in the ED. In addition, invasive procedures not performed in the ED were often performed early in the ICU admission. Other studies have reported variable procedure completion rates in the ED, as EETI rates have ranged from 13.3-30.8% [8,10,11,13], CVC rates 3.9-26%; and arterial catheter rates 0.0-14.8% [8,10,11] It is possible that some procedures may have been delayed until transfer, which may indicate that life saving therapy was delayed.

Our study highlights several important issues, namely the prolonged length of stay of critically ill patients in the ED and an apparent disparity in invasive procedures employed in the ED. Current evidence suggests that aggressive resuscitation and interpretation of physiologic data in critically ill patients is beneficial in patient outcomes, and may result in a reduction in ICU admissions. [4,13,15] It is unclear if the management provided for patents in this study was optimal, or if a reduction in the LOS or additional invasive procedures performed in the ED would have impacted on patient outcomes. Further investigation is warranted.

## Limitations

There are several limitations to our study, as this is a single center retrospective medical record review. Although we are confident that all patients admitted to the ICU during the study phase were identified, chart documentation was not complete for some of the variables examined. Despite this, we feel that the ED LOS and procedures completed which are reported are valid. Finally, the number of patients included in this study was relatively small and trauma patients, cardiac patients and patients requiring operative intervention prior to ICU admission were excluded, which does not allow interpretation of our data in this patient population.

## Conclusion

Critically ill patients are managed in the emergency department for a significant length of time. Although the majority of airway intervention occurs in the prehospital and ED setting, relatively few patients undergo invasive procedures while in the emergency department. Further research on the importance of ED LOS of critically ill patients is suggested.

## Competing interests

The authors declare that they have no competing interests.

## Authors' contributions

RG conceived and designed the study. JM reviewed and extracted patient data. Both RG and JM analyzed the data. RG prepared the manuscript, and both authors have read and approved the final manuscript.

## References

[B1] Fromm RE, Gibbs LR, McCallum WG, Niziol C, Babcock JC, Gueler AC (1993). Critical care in the emergency department: a time-based study. Crit Care Med.

[B2] Lambe S, Washington DL, Fink A, Herbst K, Liu H, Fosse JS (2002). Trends in the use and capacity of California's emergency departments, 1990-1999. Ann Emerg Med.

[B3] McCaig LF, Nawar EW (2006). National Hospital Ambulatory Medical Care Survey: 2004 emergency department summary. Adv Data.

[B4] Rivers E, Nguyen B, Havstad S, Ressler J, Muzzin A, Knoblich B (2001). Early Goal-Directed Therapy in the Treatment of Severe Sepsis and Septic Shock. N Engl J Med.

[B5] Trzeciak S, Dellinger RP, Abate NL, Cowan RM, Stauss M, Kilgannon JH (2006). Translating research to clinical practice: a 1-year experience with implementing early goal-directed therapy for septic shock in the emergency department. Chest.

[B6] Meggs WJ, Czaplijski T, Benson N (1999). Trends in emergency department utilization, 1988-1997. Acad Emerg Med.

[B7] Clark K, Normile LB (2007). Patient flow in the emergency department: is timeliness to events related to length of hospital stay?. J Nurs Care Qual.

[B8] Nelson M, Waldrop RD, Jones J, Randall Z (1998). Critical care provided in an urban emergency department. Am J Emerg Med.

[B9] Saukkonen KA, Varpula M, Rasanen P, Roine RP, Voipio-Pulkki LM, Pettila V (2006). The effect of emergency department delay on outcome in critically ill medical patients: evaluation using hospital mortality and quality of life at 6 months. J Intern Med.

[B10] Svenson J, Besinger B, Stapczynski JS (1997). Critical care of medical and surgical patients in the ED: length of stay and initiation of intensive care procedures. Am J Emerg Med.

[B11] Varon J, Fromm RE, Levine RL (1994). Emergency department procedures and length of stay for critically ill medical patients. Ann Emerg Med.

[B12] Jimenez JG, Murray MJ, Beveridge R, Pons JP, Cortes EA, Garrigos JB (2003). Implementation of the Canadian Emergency Department Triage and Acuity Scale (CTAS) in the Principality of Andorra: Can triage parameters serve as emergency department quality indicators?. CJEM.

[B13] Nguyen HB, Rivers EP, Havstad S, Knoblich B, Ressler JA, Muzzin AM (2000). Critical care in the emergency department: A physiologic assessment and outcome evaluation. Acad Emerg Med.

[B14] Bur A, Mullner M, Sterz F, Hirschl MM, Laggner AN (1997). The emergency department in a 2000-bed teaching hospital: saving open ward and intensive care facilities. Eur J Emerg Med.

[B15] Rady MY, Rivers EP, Nowak RM (1996). Resuscitation of the critically ill in the ED: responses of blood pressure, heart rate, shock index, central venous oxygen saturation, and lactate. Am J Emerg Med.

